# There Is No Structural Relationship between Nasal Septal Deviation, Concha Bullosa, and Paranasal Sinus Fungus Balls

**DOI:** 10.1100/2012/181246

**Published:** 2012-12-26

**Authors:** Tung-Lung Tsai, Ming-Ying Lan, Ching-Yin Ho

**Affiliations:** Department of Otolaryngology, Taipei Veterans General Hospital, School of Medicine, National Yang-Ming University, No. 201 Section 2, Shih-Pai Road, Taipei 112, Taiwan

## Abstract

This study aims to determine the relationship between nasal septal deviation, concha bullosa, and chronic rhinosinusitis by using a definitive pathological and simplified model. Fifty-two consecutive sinus computed tomography scans were performed on patients who received endoscopic sinus surgery and whose final diagnosis was paranasal sinus fungus balls. The incidences of nasal septal deviation and concha bullosa for patients diagnosed with paranasal sinus fungus balls among the study group were 42.3% and 25%, respectively. About 63.6% sinuses with fungus balls were located on the ipsilateral side of the nasal septal deviation, and 46.2% were located on the ipsilateral side of the concha bullosa. When examined by Pearson's chi-square test and the chi-squared goodness-of-fit test, no significant statistical difference for the presence of paranasal sinus fungus balls between ipsilateral and contralateral sides of nasal septal deviation and concha bullosa was noted (*P* = 0.292 and *P* = 0.593, resp.). In conclusion, we could not demonstrate any statistically significant correlation between the location of infected paranasal sinus, the direction of nasal septal deviation, and the location of concha bullosa, in location-limited rhinosinusitis lesions such as paranasal sinus fungal balls. We conclude that the anatomical variants discussed herein do not predispose patients to rhinosinusitis.

## 1. Introduction

Stammberger and Posawetz [1] identified mucosal contact in the middle meatus, leading to blockage of paranasal sinus drainage, as a key factor in the pathogenesis of rhinosinusitis. Since then, many anatomical abnormalities that impair sinus ventilation or lead to mucosal contact and, thus, might be expected to predispose the development of rhinosinusitis have been studied [2–14]. Among these anatomical variants, deviation of the nasal septum and the presence of a concha bullosa have been the most frequently observed alternatives [15], although the relationship between rhinosinusitis and the many reported anatomical variants still remains controversial.

It has been suggested that nasal septal deviation predisposes a patient to rhinosinusitis [4, 5, 7, 10, 13]. Calhoun et al. found the presence of septal deviation significantly associated with the diseases of ostiomeatal complex, anterior, and posterior ethmoid sinuses [4]. They noted that this effect arose only on the side to which the septum deviated. Previous studies found a high incidence of sinus diseases accompanied by septal deviation, but no apparent statistically significant difference between ipsilateral and contralateral sides in relation to the direction of the deviation [5, 7, 10, 13]. Some evidence of an association with rhinosinusitis for concha bullosa, however, has been demonstrated [2, 3, 7]. In a 800-patient sinus computed tomography (CT) scan study in 1993, Earwaker found that so-called obstructive patterns of anatomical variations exhibited an equal prevalence among patients with or without sinus disease [6]. Jones et al. (1997) compared 100 CT scans for rhinosinusitis with 100 CT scans for intraorbital disease and concluded that bony anatomical sinus variations did not appear to influence the prevalence of rhinosinusitis [8]. Saunders et al. used morphometry in 1998 to study anatomical variations among diseased and normal sinuses and arrived the same conclusion [9]. Kayalioglu et al. found that the prevalence of concha bullosa is 28.88% in sinus-diseased patients and 26.83% in non-sinus-diseased patients [11]. However, most of these study groups were composed of patients suspected to be having sinus disease but without any definitive pathological diagnosis. The effects between the variables of host immunity, causative pathogen, and anatomic variants were unclear. This makes the interpretation of those study results more complicated. Paranasal sinus fungus balls are usually unilateral and most patients are typically immunocompetent. The pathogen for this condition is unique and there is typically only one infection pathway. The characteristics of paranasal sinus fungus balls provide us with a simplified model to understand the effects of anatomical variation, such as nasal septal deviation and concha bullosa, associated with rhinosinusitis.

We conducted a radiographic study to explore a more definitive relationship between nasal septal deviation, concha bullosa, and rhinosinusitis. The specific hypothesis tested was that the sinuses draining to the narrower nasal cavity would be more vulnerable than their anatomically wider counterparts to paranasal sinus fungus balls.

## 2. Materials and Methods

Fifty-two consecutive CT scans of sinuses were obtained from the patients who underwent functional endoscopic sinus surgery, all of whose histopathological sections revealed paranasal sinus fungus balls. The CT scans were performed in both the coronal and axial projection with contiguous 4 mm sections. Only bone algorithms and bone windows were used. No contrast agent was administered to the study subjects. Patients who underwent previous sinonasal surgery were excluded from the study group. The study was approved by the hospital's Institutional Review Board (VGHIRB 201009023IC).

The presence/absence of nasal septal deviation at the level of the ostiomeatal complex on both coronal and axial sections was examined. The direction of nasal septal deviation was defined as the particular side of the nasal cavity that was compromised by the deviated nasal septum. According to Orlandi (2010), the definition of septal deviation is an angle larger than 10° at osteomeatal complex level [14].

The presence of concha bullosa was defined as the condition of pneumatization of the middle turbinate. The locations of paranasal sinus fungus balls were determined from the operative notes. While studying the relationship between the direction of nasal septal deviation and the location of paranasal sinus fungus balls, patients with paranasal sinus fungus balls in the contralateral side of the nasal septal deviation were regarded as the control group. For the investigation of the relationship between the location of concha bullosa and paranasal sinus fungus balls, patients with paranasal sinus fungus balls in the contralateral side of their concha bullosa were considered as the control group.

Statistical analysis was conducted using the statistical package for social sciences system, version 10.0 (SPSS Inc, Chicago, USA). Pearson's chi-square test and phi correlation were used to determinate the association between the direction of the nasal septal deviation and the location of paranasal sinus fungus balls. The chi-square goodness-of-fit test and phi correlation were used to determine the association between the location of concha bullosa and any paranasal sinus fungus balls.

## 3. Results

From the 52 CT scans of paranasal sinuses with histopathologically determined paranasal sinus fungus balls, 22 (42.3%) cases presented with nasal septal deviation, and 13 (25%) cases demonstrated concha bullosa. For the 22 cases revealing nasal septal deviation, 11 revealed a direction of nasal septal deviation to the right side, with the others to the left side. The locations of paranasal sinus fungus balls are summarized in Table 1. Fourteen paranasal sinuses with fungus balls were detected within the ipsilateral side of the nasal septal deviation, and 9 paranasal sinuses with fungus balls were located on the contralateral side of the nasal septal deviation. There was, however, no statistical difference between ipsi- and contralateral sides for location of fungus balls when examined by Pearson's chi-square test (*P* = 0.292 > 0.05), although the phi value did reveal a rather weak positive correlation (Φ = 0.220) between the direction of nasal septal deviation and the location of paranasal sinus fungus balls.

For cases presenting concha bullosa upon a CT scan of the paranasal sinus, the locations of paranasal sinus fungus balls are summarized in Table 2. There were 6 paranasal sinuses with fungus balls located on the ipsilateral side of the concha bullosa. Eight paranasal sinuses with fungus balls were found on the contralateral side of the concha bullosa. When tested by chi-square goodness-of-fit test and phi correlation, the *P* value was 0.593 and phi value was −0.091, thus, there appeared to be no significant statistical difference. Among the 22 patients with deviated nasal septum, 6 were noted to demonstrate concha bullosa on inspection of the coronal CT scans. In 3 patients each, concha bullosa was found on the ipsilateral and contralateral sides of the nasal septal deviation.

## 4. Discussion

Our data revealed that the prevalence of nasal septal deviation is 42.3% among patients with paranasal sinus fungus balls. This is higher than that reported by Jones et al. [8] (24% for normal and sinus-diseased groups) and Kayalioglu et al. in 2000 [11] (22.2% for sinus-disease patients and 12% for non-sinus-diseased patients), but congruent to the findings of Earwaker (1993) [6] (44% on the basis of patients' CT for functional endoscopic sinus surgery evaluation) and Pérez-Piñas et al. (2000) [15] (58% amongst patients with sinus disease). Concha bullosa appeared in 25% of patients who revealed paranasal fungus balls. This result parallels many other authors' reports for diseased groups (17.4%–37%) [3–8, 11, 15, 16]. As regards the relationship between the direction of the nasal septal deviation and the location of paranasal sinus fungus balls, our results suggest no statistically significant difference between ipsilateral and contralateral sides of the septal deviation. This result agrees with the results of Yousem et al. [5], Elahi et al. [7], and Elahi and Frenkiel [10] and Yasan et al.[13] but contradicts the results of Calhoun et al. [4]. Elahi and Frenkiel attributed this finding to abnormalities of the middle turbinate and lateral nasal wall in the contralateral side of septal deviation [10]. From our observations, however, only 13.6% (3/22) of the cases of nasal septal deviation presented with concha bullosa on the contralateral side of the septal deviation.

Our data also revealed no statistically significant difference between ipsilateral and contralateral sides of concha bullosa as regards the likelihood of the presence of sinus fungus balls. Bolger et al. [3] (1991) reported “pneumatization of the inferior bulbous portion of the middle turbinate” for 35.3% of patients presenting with a chronic sinus complaint, but only 13.9% of non-sinus-diseased patients with statistically significant difference between the 2 groups. Although these authors did not clarify the relationship between the presence of concha bullosa and the affected sinuses, they concluding that pathogenicity of this anatomic variation may need to be considered on a patient-by-patient basis. We selected patients with paranasal sinus fungus balls, which typically display the character of a single sinus infection, in order to provide us clearer evidence of the relationship between rhinosinusitis and the presence of concha bullosa.

Stammberger [17] (1985) suggested that the anatomical variations over the middle meatus may be attributable to the stenosis of the ostiomeatal complex, resulting in a low pH environment for the growth of fungal hyphae. As more data have been collected, this hypothesis has been challenged. For location-limited rhinosinusitis lesions such as paranasal sinus fungus balls, we are still unable to determine any statistically significant correlation between the location of the infected paranasal sinus and the direction of the associated nasal septal deviation (if present), or with the location of concha bullosa. It is possible that the narrower side of the nasal cavity could be responsible for ostiomeatal complex obstruction, leading to rhinosinusitis and thus, providing a hypoxic environment in which fungal spores are able to grow. However, our data cannot support Stammberger's hypothesis.

We conclude from our findings that (1) it is possible for the pathogenesis of paranasal fungus balls to be different from that of bacterial rhinosinusitis, that is, the ostiomeatal complex may be sufficiently patent so as to provide an entrance for fungal spores in the process of fungus ball formation and (2) just as other authors have concluded, the anatomical variants discussed above do not necessarily predispose a patient to rhinosinusitis. Further, the occurrence of certain definite mucosal changes, resulting in impaired ciliary function, may be a more important predisposing factor for rhinosinusitis, as evidenced by the finding of Passàli et al. [18], who, in 1999, reported that the nasal mucociliary transport time did not vary for patients suffering from nasal septal deviations, but did vary for chronic rhinosinusitis patients. We emphasize that the number of cases we studied wes somewhat limited, and our data should be interpreted with some caution although we stress that, to the best of our knowledge, this is the first study to examine the relationship between the anatomical variation of the nasal region and the location of paranasal sinus fungus balls. We believe that the study of location-limited infectious sinus disease such as paranasal fungus balls will simplify the variables of host immunity and causative pathogen in rhinosinusitis, thereby providing us with a simple model to comprehend the pathogenesis of rhinosinusitis or fungal sinusitis.

## Figures and Tables

**Table 1:** The direction of nasal septal deviation and the location of paranasal fungus balls.

The direction of nasal septal deviation	The location of paranasal fungus balls		
	Lt maxillary sinus	Rt maxillary sinus	Bil sphenoid sinus
Right (Rt)	4	7	0
Left (Lt)	6	4	1

**Table 2:** The location of concha bullosa and the location of paranasal fungus balls.

The location of concha bullosa	The location of paranasal fungus balls		
	Lt maxillary sinus	Rt maxillary sinus	Bil sphenoid sinus
Right	3	1	0
Left	2	5	1
Bilateral (Bil)	1	1	0

## References

[1] Stammberger H, Posawetz W (1990). Functional endoscopic sinus surgery. Concept, indications and results of the Messerklinger technique. *European Archives of Oto-Rhino-Laryngology*.

[2] Lloyd GAS (1990). CT of the paranasal sinuses: study of a control series in relation to endoscopic sinus surgery. *Journal of Laryngology and Otology*.

[3] Bolger WE, Butzin CA, Parsons DS (1991). Paranasal sinus bony anatomic variations and mucosal abnormalities: CT analysis for endoscopic sinus surgery. *Laryngoscope*.

[4] Calhoun KH, Waggenspack GA, Simpson CB, Hokanson JA, Bailey BJ (1991). CT evaluation of the paranasal sinuses in symptomatic and asymptomatic populations. *Otolaryngology*.

[5] Yousem DM, Kennedy DW, Rosenberg S (1991). Ostiomeatal complex risk factors for sinusitis: CT evaluation. *Journal of Otolaryngology*.

[6] Earwaker J (1993). Anatomic variants in sinonasal CT. *Radiographics*.

[7] Elahi MM, Frenkiel S, Fageeh N (1997). Paraseptal structural changes and chronic sinus disease in relation to the deviated septum. *Journal of Otolaryngology*.

[8] Jones NS, Strobl A, Holland I (1997). A study of the CT findings in 100 patients with rhinosinusitis and 100 controls. *Clinical Otolaryngology and Allied Sciences*.

[9] Saunders NC, Birchall MA, Armstrong SJ, Killingback N, Singh GD (1998). Morphometry of paranasal sinus anatomy in chronic rhinosinusitis: a pilot study. *Archives of Otolaryngology*.

[10] Elahi MM, Frenkiel S (2000). Septal deviation and Chronic Sinus disease. *American Journal of Rhinology*.

[11] Kayalioglu G, Oyar O, Govsa F (2000). Nasal cavity and paranasal sinus bony variations: a computed tomographic study. *Rhinology*.

[12] Stallman JS, Lobo JN, Som PM (2004). The incidence of concha bullosa and its relationship to nasal septal deviation and paranasal sinus disease. *American Journal of Neuroradiology*.

[13] Yasan H, Doğru H, Baykal B, Doüner F, Tüz M (2005). What is the relationship between chronic sinus disease and isolated nasal septal deviation?. *Otolaryngology*.

[14] Orlandi RR (2010). A systematic analysis of septal deviation associated with rhinosinusitis. *Laryngoscope*.

[15] Pérez-Piñas I, Sabaté J, Carmona A, Catalina-Herrera CJ, Jiménez-Castellanos J (2000). Anatomical variations in the human paranasal sinus region studied by CT. *Journal of Anatomy*.

[16] Bayis U, Dursun E, Islam A (2005). Is septoplasty alone adequate for the treatment of chronic rhinosinusitis with septal deviation?. *American Journal of Rhinology*.

[17] Stammberger H (1985). Endoscopic surgery for mycotic and chronic recurring sinusitis. *The Annals of Otology, Rhinology & Laryngology*.

[18] Passàli D, Ferri R, Becchini G, Passàli GC, Bellussi L (1999). Alterations of nasal mucociliary transport in patients with hypertrophy of the inferior turbinates, deviations of the nasal septum and chronic sinusitis. *European Archives of Oto-Rhino-Laryngology*.

